# The Building Blocks of Implementation Frameworks and Models in Primary Care: A Narrative Review

**DOI:** 10.3389/fpubh.2021.675171

**Published:** 2021-08-03

**Authors:** Ine Huybrechts, Anja Declercq, Emily Verté, Peter Raeymaeckers, Sibyl Anthierens

**Affiliations:** ^1^Department of Family Medicine and Population Health, University of Antwerp, Antwerp, Belgium; ^2^Department of Family Medicine and Chronic Care, Free University of Brussels, Brussels, Belgium; ^3^LUCAS – Centre for Care Research and Consultancy & CESO – Centre for Sociological Research, Catholic University of Leuven, Leuven, Belgium

**Keywords:** primary care interventions, implementation, implementation frameworks, implementation models, implementation process, implementation science

## Abstract

**Background:** Our aim is to identify the core building blocks of existing implementation frameworks and models, which can be used as a basis to further develop a framework for the implementation of complex interventions within primary care practices. Within the field of implementation science, various frameworks, and models exist to support the uptake of research findings and evidence-based practices. However, these frameworks and models often are not sufficiently actionable or targeted for use by intervention designers. The objective of this research is to map the similarities and differences of various frameworks and models, in order to find key constructs that form the foundation of an implementation framework or model that is to be developed.

**Methods:** A narrative review was conducted, searching for papers that describe a framework or model for implementation by means of various search terms, and a snowball approach. The core phases, components, or other elements of each framework or model are extracted and listed. We analyze the similarities and differences between the frameworks and models and elaborate on their core building blocks. These core building blocks form the basis of an overarching model that we will develop based upon this review and put into practice.

**Results:** A total of 28 implementation frameworks and models are included in our analysis. Throughout 15 process models, a total of 67 phases, steps or requirements are extracted and throughout 17 determinant frameworks a total of 90 components, constructs, or elements are extracted and listed into an Excel file. They are bundled and categorized using NVivo 12© and synthesized into three core phases and three core components of an implementation process as common elements of most implementation frameworks or models. The core phases are a development phase, a translation phase, and a sustainment phase. The core components are the intended change, the context, and implementation strategies.

**Discussion:** We have identified the core building blocks of an implementation framework or model, which can be synthesized in three core phases and three core components. These will be the foundation for further research that aims to develop a new model that will guide and support intervention designers to develop and implement complex interventions, while taking account contextual factors.

## Introduction

Initiating and sustaining change within primary care is challenging (1). Most change that is introduced in primary care takes the form of a complex intervention, meaning that it involves concepts that are rather difficult to measure and its components are often interconnected (2, 3). Nowadays, there are increased efforts to shift toward a more patient-centered approach (1), as this proves to improve disease outcomes and quality of life (4). However, such a shift highly challenges current primary care practices and there is therefore no consensus on how to best implement it (5). This indicates a gap between scientific evidence and actual practice: an evidence-to-practice gap (3). This can also be referred to as “*the black box of knowledge translation”* (6), meaning that much uncertainty exists about understanding why evidence-based practices do not find their way into real world settings and investigating how such complexities can best be approached.

Concrete initiatives and strategies for implementation often do not match with targeted problems (7). In the end, too much is expected from practitioners' ability and goodwill to consult, interpret, and adapt their practices in line with best evidence of research findings (8). The World Health Report 2008^1^ stated that “*providing a sense of direction to health systems requires a set of specific and context-sensitive reforms that respond to the health challenges of today and prepare for those of tomorrow*.” It is thus key to carefully define specific interventions that aim to transform current practices, while at the same time tailoring them to local circumstances (9, 10). To do this, it is essential to gain insight in the process of implementation as well as in potential barriers and facilitators that might hinder or support the implementation process. This is studied in the field of implementation science, which is “*the scientific study of methods to promote the systematic uptake of research findings and other evidence-based practices into routine practice* *(**11**).”* The goal of implementation science is to close the gap between evidence-based practices and the extent to which research findings are integrated into real world settings and practices (3, 12).

Within the field of implementation science, many theories, models and frameworks have been created by various disciplines. Moreover, there is a variety of guidelines and tools aimed at facilitating the integration of knowledge of implementation science into either the development or the initiation of interventions and how to document this process. Examples are the ImpRes tool (13), NCEC Implementation Guide & Toolkit for National Clinical Guidelines (14), RNAO Toolkit: Implementation of Best Practice Guidelines (2^nd^ ed). (15), STaRi Standards for Reporting Implementation Studies (16) and Implementation Research Logic Model (17). However, the landscape of implementation science is rather difficult to navigate, as there is a lack of guidance for selecting theories, frameworks, models, or tools that best fit specific implementation objectives (18). A first step toward a better comprehension of such guidance on implementation efforts and to focus on concepts that are more meaningful to the actors in the field, is to gain better understanding in the common thread throughout the wide variety of models and frameworks that form the basis of such tools.

Current approaches to guide the implementation process are mainly characterized by a single-discipline, medical perspective in which a limited number and types of barriers are taken into account (19). This is insufficient to provide a deeper understanding of implementation success or failure or to increase the chance of success of the implementation (20). Existing frameworks and models tend to incorporate a selection of barriers, but do not allow to give more guidance about their validity or relative importance in specific contexts (20). Moreover, many frameworks and models remain very abstract and fall short in giving concrete guidance for intervention designers on how to navigate the implementation process (21). As many of such frameworks or models remain untested, this again questions their operability (7).

Therefore, an overarching framework is needed that provides both an explanatory approach (3), but also allows to prioritize those variables that are essential to achieve implementation success (22). This means that such a framework should provide a pathway that clarifies the core phases and steps throughout an implementation process and that highlight the core constructs that, within each phase, need to be defined, acted upon, and reflected upon. These phases and constructs should be accessible and meaningful to actors that will conduct implementation efforts. It is key for such framework to transcend disciplines and to bundle insights from different approaches (7).

This research is a first step in the development of a generic framework that incorporates such an approach. We therefore looked into existing theories, models, and frameworks from implementation science and combined insights across various disciplines. The similarities and differences between various frameworks informed us about the main building blocks of such frameworks and about how and why they differ. In doing this, we were guided by a rather broad research question: “*What are the main components of implementation frameworks and models in order to structure and guide implementation processes?”* This resulted into the identification of core building blocks that form a common thread throughout implementation models and frameworks. Such synthesis will in future research help to develop an overarching model that puts forward clear and meaningful constructs for intervention designers, and that provides both a pathway as well as an explanatory structure to define, act, and reflect upon each component of a complex intervention.

## Methods

To determine the building blocks of an overarching implementation framework, we conducted a literature review. Various disciplines were represented in the included literature, for which the initial search had been conducted by a multi-disciplinary team of medical researchers, sociologists, social work, and agogic sciences. We opted for a narrative review, which can be defined as “*comprehensive narrative syntheses of previously published information* (23)” and which helps to “*pull many pieces of information together into a readable format* (23).” This reviewing technique is particularly helpful for grasping a broad perspective on a topic; it enables us to transcend a purely medical view on primary care and incorporate other perspectives such as social welfare. Moreover, since the field of implementation science is rather fragmented and consists of a wide range of sources, it requires a wider scoping (24). Instead of focusing on a more rigor methodology to answer a very specific, narrowly-focused research question (24), a narrative review allows for interpretation and critique, aiming to deepen the overall understanding of the subject specifically targeted at our problem (24). This corresponds to our goal to identify and possibly simplify the complexities of implementing an intervention by extracting the core phases and components that are common in most models. According to Green, Johnson and Adams (23), a successful narrative review synthesizes available evidence in relation to a topic and present it in a structured way, conveying a clear message. Our aim is thus to provide an overview of existing implementation frameworks and models and to analyze how they are structured and build.

Our initial search started with articles that were key in identifying other models and frameworks: Nilsen (25) which categorized many frameworks and models and Damschroder et al. (26) which provided a list of references on which the consolidated framework for advancing implementation science was based. Our search continued with consulting the three databases PubMed, Web of Science, and Google Scholar, which are most commonly used in this type of literature. The key words that were used are listed in Table 1. Article titles and abstracts were screened for references about a specific framework, model, or theory for implementation, followed by an additional search for theoretical papers on these frameworks, models, or theories. Subsequently, the search terms were adapted and redefined based upon our findings, thus creating an iterative process that ensures covering literature in a comprehensive way (27). Also, a snowball approach was used and additional literature was found in the references of the papers.

**Table 1:** Overview of the process of searching articles.

**1) References in key articles**		
Nilsen (25) Damschroder et al. (26)	Provides a categorization of frameworks and models and gives many examples of each type. Provides a list of references on which the consolidated framework for advancing implementation science was based.	
**2) Database search**		
**Databases** **(Between 2000 and May 2020)**	**List of search terms**	
PubMed Web of Science Google Scholar	“Primary care” or “primary care interventions” or “health services” AND “implementation framework” or “implementation model” or “implementation science”	
**3) Adaptation of search terms based on findings**		

Articles were searched for and consulted between October 2019 and May 2020. They were mostly published between the years 2000 and 2020, but we did include some older source material if a model or framework was considered to be relevant (e.g., the paper was often referred to by other relevant articles). All articles were available as full text in English. We looked for articles which primarily consisted of a theoretical elaboration (and/or application) of a specific framework or model. Frameworks and models that were highly targeted toward a single case or strategy were excluded, as they were difficult to generalize for overall primary care settings.

To compare and analyze the frameworks and models, they were listed and classified according to Nilsen's (25) categorization (see: Table 2). We built our analysis upon process models and determinant frameworks, as they allowed to extract clear steps, actions, barriers, and facilitators that can be transformed into guidance for intervention designers, which was the main aim of our research. For additional understanding of the component evaluation that came up in several models and frameworks, we also looked into three evaluation frameworks. Several classic theories [e.g., Theory of Diffusion (28)] and implementation theories [e.g., Normalization Process Theory (29)] were initially identified, but were not included in our analysis as their approach and structure did not match with our goal to extract clear building blocks of an implementation process that could be used to reconstruct a generic framework.

**Table 2:** Five categories of theories, models and frameworks used in implementation science.

**Category**	**Description**
Process models	Specify steps (stages, phases) in the process of translating research into practice, including the implementation and use of research. The aim of process models is to describe and/or guide the process of translating research into practice. An action model is a type of process model that provides practical guidance in the planning and execution of implementation endeavors and/or implementation strategies to facilitate implementation.
Determinant frameworks	Specify types (also known as classes or domains) of determinants and individual determinants, which act as barriers and enablers (independent variables) that influence implementation outcomes (dependent variables). Some frameworks also specify relationships between some types of determinants. The overarching aim is to understand and/or explain influences on implementation outcomes, e.g., predicting outcomes or interpreting outcomes retrospectively
Classic theories	Theories that originate from fields external to implementation science, e.g., psychology, sociology, and organizational theory, which can be applied to provide understanding and/or explanation of aspects of implementation
Implementation theories	Theories that have been developed by implementation researchers (from scratch or by adapting existing theories and concepts) to provide understanding and/or explanation of aspects of implementation
Evaluation frameworks	Specify aspects of implementation that could be evaluated to determine implementation success

*Categorization and definitions by Nilsen (25)*.

To analyze, all relevant frameworks and models were listed in an Excel file, with an overview of how they were constructed. For process models, their main phases (steps, stages) were listed, together with relevant details or components within the process they described. For determinant frameworks, the main components (constructs, elements) were listed, together with any details or further clarification about each of the components described. The first step to analyze was to bundle each of the phases or components that had a similar approach or meaning. This was done by the main researcher and validated by the three senior researchers. An overarching concept was appointed to each group of concepts. Then, NVivo 12© was used to structure the main themes and concepts and to analyze their similarities and differences. The overarching concepts were entered as the main nodes in NVivo 12©, whereby details or explanation about each concept from the different models and frameworks were again coded when we noticed overlap with approaches from different frameworks or models. By structuring the phases and components this way and by analyzing the details that were given for each component, we could synthesize it into core building blocks.

## Results

Fifteen process models and 17 determinant frameworks were identified. Four models had characteristics of both a process model as well as a determinant framework: the Conceptual Model of Evidence-Based Practice Implementation in Public Service Sectors (22), the Consolidated Framework for Implementation Research (26), The Ottawa Model of Health Care Research (30) and the Generic Implementation Framework (19). The frameworks or models focus on various domains. They were either developed specifically to apply within a certain research domain or development was based upon a single discipline. Table 3 gives an overview of the process models and determinant frameworks that were incorporated in our analysis per research domain. As we have only included English literature, this is largely represented in the geographical distribution of the included literature: 18 articles derive from authors affiliated with institutions located in the United States of America, 5 in the United Kingdom, 2 in Canada, 1 in Australia (in collaboration with a Spanish and Portuguese institution), 1 in Ireland, and 1 in Sweden.

**Table 3:** Overview of process models and determinant frameworks per domain.

**Domain**	**Process models**	**Determinant frameworks**
Implementation science or interdisciplinary	Consolidated Framework for Implementation Research (CFIR) (26), Advancing Understanding of Mechanism of Change in Implementation Science (31), Quality Implementation Framework (32), Ottawa Model of Health Care Research (30), Generic Implementation Framework (GIF) (19)	Consolidated Framework for Implementation Research (CFIR) (26), Integrated Promoting Action Research in Health Services Framework (i-PARiHS) (33), Understanding User Context Framework for Knowledge Translation (34), Interdisciplinary Conceptual Framework of Clinicians' Compliance with Evidence-based Guidelines (35), A Practical, Robust Implementation and Sustainability Model (PRISM) (36), Determinants and Consequences of Implementation Effectiveness (37), Conceptual Framework (3), Generic Implementation Framework (GIF) (19)
Medical sciences	Medical Research Council guidance (38), A Model for Large Scale Knowledge Translation (39)	Four levels of change for improving quality (40), Translating Research into Practice (41), Barrier Assessment (20)
Nursing	IOWA Model (42), Stetler Model of Research Utilization (43), ACE Star Model of Knowledge Transformation (44)	
Pharmacy	Active Implementation Frameworks (45)	
Public health or prevention research	The NCCDPHP Knowledge to Action Framework for Public Health (46), Research Utilization Model (modified from Rogers) (47)	Ecological Framework—Interactive Systems Framework for Dissemination and Implementation (48)
Organization research or service innovations	Organizational model for transformational change in health care systems (49)	Conceptual Model for Considering the Determinants of Diffusion, Dissemination, and Implementation of Health Service Delivery and Organization (50)
Social and behavioral sciences		Theoretical Domains Framework (V2.0) (51)
Social work	Conceptual Model of Evidence-Based Practice Implementation in Public Services Sectors (22)	Conceptual Model of Evidence-Based Practice Implementation in Public Services Sectors (22), the CAIMeR Theory (52)

Through analysis of both process models and determinant frameworks, we were able to grasp (1) a logical pathway in which different actions need to be taken in order to successfully implement a complex intervention, and (2) the main building blocks of which the intervention consists.

Table 4 gives an overview of the 15 process models with the main phases, steps, or requirements we could detract in each model (67 in total) and Table 5 gives an overview of the 17 determinant frameworks and the main components, constructs, or elements that were put forward in these frameworks (90 in total). This served as a basis on which we detracted the common thread in each of these models and frameworks. We identified three main phases which most models have in common: a development phase, a translation phase, and a sustainment phase. Throughout all process models, 54 phases, steps, or requirements could directly be linked to these three phases. We also identified three main components: the intended change, the context, and the implementation strategies. A total of 67 components, constructs, or elements from all determinant frameworks could be directly linked to these three main components (see: Table 5). Thirteen components from 10 different process models could also be linked to these three main components (see: Table 4). Additionally, 17 components from 10 different determinant frameworks could indirectly be linked to the three main components as either outcomes, actors or processes (see: Table 5), leaving only 6 components that were not linked to the core phases and components we identified.

**Table 4:** Overview of process models with their main phases, steps, or requirements.

	**Framework**	**Phases/steps/requirements**
Models who distinguish between phases of the implementation process	Medical Research Council guidance, Craig et al. (38)	Development Feasibility and piloting *Evaluation* Implementation
	Conceptual Model of Evidence-Based Practice Implementation in Public Service Sectors, Aarons et al. (22)	Exploration Adoption decision/Preparation Active implementation Sustainment
	Consolidated Framework for Implementation Research (CFIR), Damschroder et al. (26)	Planning Engaging Executing *Reflecting and evaluating*
	NCCDPHP Knowledge to Action Framework for Public Health, Wilson et al. (46)	Research phase Translation phase Institutionalization phase
	Research Utilization Model (modified from Rogers), Davis et al. (47)	Stage 0. Research Development *Stage 1. Dissemination* Stage 2. Intent to adopt Stage 3.a Implementation Stage 3.b Adaptation Stage 4. Institutionalization Stage 5. Diffusion and replication
	Active Implementation Frameworks, Blanchard et al. (45)	Exploration Installation Initial implementation Full implementation
	Stetler Model of Research Utilization, Stetler (43)	Phase 1: Preparation Phase 2: Validation Phase 3: Comparative Evaluation Phase 4: Decision making Phase 5: Translation/application Phase 6: Evaluation
	Generic Implementation Framework (GIF), Moullin et al. (19)	Pre-implementation Process of implementation Post-implementation
Action models with a step-wise approach	ACE Star Model of Knowledge Transformation, Stevens (44)	Discovery Research Evidence Summary Translation to Guidelines Practice Integration *Process, Outcome Evaluation*
	A model for large scale knowledge translation, Pronovost et al. (39)	1. Summarize the evidence *2*. Identify local barriers to implementation *3. Measure performance* *4*. Ensure all patients receive the interventions
	Advancing understanding of mechanism of change in implementation science, Lewis et al. (31)	*Step 1: Specifying implementation strategies* *Step 2: Generating strategy-mechanism linkages* *Step 3: Identifying proximal and distal outcomes* *Step 4: Articulating effect modifiers*
	Organizational model for transformational change in health care systems, Lukas et al. (49)	Impetus to Transform *Leadership* Improvement Initiatives Alignment Integration
	The ottawa model of health care research, Logan et al. (30)	1. Assess: Practice environment, potential adopters, evidence-based innovation 2. Monitor: Transfer strategies, adoption 3. Evaluate: Outcomes
	Quality Implementation Framework, Meyers et al. (32)	1. Initial considerations regarding the host setting *2*. Creating a structure for implementation *3*. Ongoing structure once implementation begins *4*. Improving future applications
	IOWA Model, Doody and Doody (42)	1. Selection of a topic *2*. Forming a team *3*. Evidence retrieval *4*. Grading the evidence *5*. Developing an EBP Standard *6*. Implement the EBP *7. Evaluation*

*The underlined phases/steps/requirements are those that are directly incorporated into the three main phases we put forward as common thread in these models.

*The phases/steps/requirements in italics are linked to the three main components as described in Framework components.

**Table 5:** Overview of determinant frameworks with their main components, constructs, or elements.

**Determinant framework**	**Components/constructs/elements**
Consolidated Framework for Implementation Research (CFIR), Damschroder et al. (26)	Intervention Characteristics *Individuals involved* Inner setting Outer setting *Process*
Integrated Promoting Action Research in Health Services Framework (i-PARiHS), Stetler et al. (33)	Evidence/Evidence and EBP characteristics (revised version) Context/Contextual readiness for targeted EBP implementation (revised version) Facilitation *Successful implementation (revised version)*
CAIMeR theory, Blom and Morén (52)	Contexts *Actors* Interventions *Mechanisms* *Results*
Barrier assessment, Cochrane et al. (20)	Cognitive-behavioral barriers Attitudinal or rational-emotional barriers Professional barriers Barriers embedded in the guidelines or evidence Patient barriers Support or resources System and process barriers
Ecological Framework—Interactive Systems Framework for Dissemination and Implementation, Durlak and DuPre (48)	Community level factors Provider characteristics Characteristics of the innovation Factors relevant to the prevention delivery system Organizational capacity Factors related to the prevention support system
Conceptual model for considering the determinants of diffusion, dissemination, and implementation of health service delivery and organization, Greenhalgh et al. (50)	The innovation System antecedents for innovation System readiness for innovation *Adopter* Assimilation *Implementation process* *Linkage* Outer context Communication and influence Diffusion and dissemination
Understanding user context framework for knowledge translation, Jacobson et al. (34)	*The user group* The issue The research The researcher-user relationship Dissemination strategies
The interdisciplinary conceptual framework of clinicians' compliance with evidence-based guidelines, Gurses et al. (35)	System characteristics Provider characteristics Guideline characteristics Implementation characteristics
Four levels of change for improving quality, Ferlie and Shortell (40)	Individual change Group/team change Organizational change Larger system/environment change
A practical, robust implementation and sustainability model (PRISM), Feldstein and Glasgow (36)	Program (Interventions) External environment Implementation and sustainability infrastructure *Recipients*
Translating research into practice, Bradley et al. (41)	Top-down support Leadership Credibility of evidence-based practice Organizational culture Coordination of different stakeholders Intervention infrastructure Dissemination Diffusion
Determinants and consequences of implementation effectiveness, Klein and Sorra (37)	Climate for implementation Skills Incentives and disincentives Absence of obstacles Innovation values fit Commitment Strategic accuracy of innovation adoption *Implementation effectiveness* *Innovation effectiveness*
Conceptual framework, Lau et al. (3)	External context Organization Professional Intervention
Generic Implementation Framework (GIF), Moullin et al. (19)	Innovation Context domains Strategies Factors *Evaluations*
The ottawa model of health care research, Logan et al. (30)	Practice environment *Potential adopters* Evidence-based innovation Transfer strategies Adoption *Outcomes*
Theoretical domains framework (v2.0), Atkins et al. (51)	Knowledge, skills, social/professional role and identity, beliefs about capabilities, optimism, beliefs about consequences, reinforcement, intentions, goals, memory, attention and decision processes, environmental context andresources, social influences, emotion, behavioral regulation
Conceptual model of evidence-based practice implementation in public service sectors, Aarons et al. (22)	Outer context Inner context *Interconnections*

*The underlined components/constructs/elements are those that are directly incorporated into the three main components we put forward as common thread in these frameworks.

*The components/constructs/elements in italics are linked to the three main components in Framework components, as either outcomes or evaluation (linked to intended change), actors (linked to context) or process (linked to strategies).

The three core phases we identified simplify the implementation process and are relevant to distinguish between different actions that need to be taken at different points in the process. The three components we identified are the core building blocks of the intervention: the way these components are approached and interact with each other will determine implementation success. Therefore, intervention designers need to reflect on how to approach each of the components within each of the phases.

### Phases of an Implementation Process

To examine different phases of an implementation process, we look at process models, as defined by Nilsen (25). Such models are built to make sense of the different phases or steps of the implementation process of an intervention (25). The goal is to construct and clarify a “logical pathway” that can give concrete guidance for intervention designers. Many models were designed with the objective of translating research evidence into real world practice (33, 39, 44, 46) or the so called shift from knowledge to action [cfr. Wilson et al. (46)]. They tend to depart from an evidence base that needs to be translated into real world settings (33, 39, 44, 46). Other models incorporate a research development phase (38, 46, 47) in which best practices are still to be defined.

We find variation among models as to what is viewed as the main process of implementation. In some models such process takes the form of a stepwise approach to ensure successful implementation of an intervention (30–32, 42, 49, 51). Nilsen (25) calls these action models. They are built upon critical steps or phases that need to be followed or focused upon in order to reach successful implementation. These main phases or steps can either be aimed at the implementation process itself (31, 32) or at the process of using research to initiate change (42, 51). In such models, key drivers or components tend to be highlighted that are necessary for change (33, 49) and/or they have a thorough focus on those strategies that will lead to sustainable change, which is referred to as general implementation strategies (31), transfer strategies (30), capacity-building strategies (32) et cetera.

Another approach for describing a process is to have models differentiate between the main phases of how implementation efforts takes form, in order to make sense of the implementation process itself (22, 38, 43, 45–47). These models describe similar phases. They distinguish between either a development (38, 47), preparation (43) or exploration phase (22, 45), a pre-adoption phase [such as piloting (38), installation (45), or the intent/decision to adopt (22, 43, 47)], an actual implementation- (22, 38, 45, 47) or translation phase (43, 46) and a sustainment (22) or institutionalization (46, 47) phase. We reduce these models to three core phases: a development phase, a translation phase and a sustainment phase—as depicted in Table 6. This is a simplification that is relevant for intervention designers and practitioners, as these phases make most sense to them as distinct phases that require other types of action from them.

**Table 6:** Overview of process models in relation to a development phase, translation phase and sustainment phase.

	**Framework**	**Development phase**	**Translation phase**	**Sustainment phase**
Models who distinguish between phases of the implementation process	Medical research council guidance, Craig et al. (38)	Development	Feasibility and piloting Implementation	–
	Conceptual model of evidence-based practice implementation in public service sectors, Aarons et al. (22)	Exploration Adoption Decision/Preparation	Active implementation	Sustainment
	Consolidated Framework for Implementation Research (CFIR), Damschroder et al. (26)	Planning Engaging	Executing	–
	NCCDPHP knowledge to action framework for public health, Wilson et al. (46)	Research phase	Translation phase	Institutionalization phase
	Research utilization model (modified from Rogers), Davis et al. (47)	Research Development Intent to adopt	Implementation Adaptation	Institutionalization Diffusion and replication
	Active implementation frameworks, Blanchard et al. (45)	Exploration	Installation Initial implementation	Full implementation
	Stetler model of research utilization, Stetler (43)	Preparation validation comparative evaluation decision making	Translation/application	–
	Generic Implementation Framework (GIF), Moullin et al. (19)	Pre-implementation	Process of implementation	Post-implementation
	ACE star model of knowledge transformation, Stevens (44)	Discovery Research Evidence Summary Translation into guidelines	Practice integration	–
	A model for large scale knowledge translation, Pronovost et al. (39)	Summarize the evidence Identify local barriers to implementation	–	Ensure all patients receive the interventions
Action models with a step-wise approach	Advancing understanding of mechanism of change in implementation science, Lewis et al. (31)	–	–	–
	Organizational model for transformational change in health care systems, Lukas et al. (49)	Impetus to Transform	Improvement Initiatives Alignment	Integration
	The ottawa model of health care research, Logan et al. (30)	Assess (practice environment, potential adopters, evidence-based innovation)	Monitor (transfer strategies, adoption)	-
	Quality implementation framework, Meyers et al. (32)	Initial considerations regarding the host setting Creating a structure for implementation	Ongoing structure once implementation begins	Improving future applications
	IOWA model, Doody and Doody (42)	Selection of a topic Forming a team Evidence retrieval Grading the evidence Developing an EBP standard	Implement the EBP	–

#### Development Phase

The development phase is the initial phase in which preparatory activities are conducted in order to successfully introduce the intervention. In the different models, various elements are considered to be relevant in this initial phase, which leads to a variety of actions that can be taken to prepare for and develop an intervention. Overall, the development phase comprises:

1. Synthesizing or collecting research evidence on which an intervention can be based;
2. Exploring the host setting;
3. Considering the overall fit of an intervention within a particular setting;
4. Ensuring readiness and intend to adopt the intervention.

Most models require that intervention designers synthesize existing evidence (38, 39, 42, 44), or that they conduct their own (discovery) research (44, 46). This will lead to either a theory (38), approach or practice (46), or research findings that can be translated into an evidence based practice (EBP) standard (42) or guidelines (44). Other models have a different focus and depart from the idea of planning (26) for an intervention or a more general exploration phase (22, 45). This is less focused on research translation and more intended to gain awareness of an issue (22), and to explore practices and implementation strategies that might respond to this issue (22). Exploration could also refer to assessing the feasibility of implementation intentions or examining the readiness of the setting in which an intervention should take place (45). This is in line with Meyers, Durlak and Wandersman (32) who mention the importance of “*initial considerations regarding the host setting*,” which refers to exploring whether there is a fit between an intervention and the host setting.

The fit between an intervention and the host setting (32, 45) can be linked to the need to asses contextual factors in this initial development phase (10, 53). The Ottawa Model of Health Care Research refers to “*assess”* as a first step, which means that the implementation environment, potential adopters, and the evidence-based innovation itself have to be examined (30). This is relevant when trying to assess the feasibility and compatibility of the intervention within a specific context. Pronovost et al. (39) mention a barrier assessment, which is a similar approach as the Conceptual Model of Evidence-Based Practice Implementation in which much emphasis is placed on mapping various hindering and promoting context variables in order to increase implementation success (22). These models recognize the importance of scanning contextual variables to identify barriers and facilitators that will affect implementation efforts.

Lastly, some models incorporate the decision or intend to adopt as a key element of the initial phase (22, 30, 43, 47). Lukas et al. (49) refer to this as the “*impetus to transform,”* which indicates that the decision to adopt a certain intervention is affected by various elements (22). This relates back to overall practitioner readiness (45), and the fit between the intervention and the setting in which it will be implemented (32, 43). According to the Quality Implementation Framework, a key step in the initial phase is also to create a structure for implementation (32). This can mean having a plan for implementation (32), but also to form a team that is dedicated to ensure implementation of an intervention (32, 42). The CFIR also recognizes the importance of engaging different actors that are involved in the intervention and views it as one of the core activities in the first phases of developing an intervention (26).

#### Translation Phase

Many frameworks refer to an implementation phase (22, 38, 42, 45, 47). It can also be called executing (26), adoption (30), improvement initiatives (49), or practice integration (44). Following the definition of Blanchard et al. (45), the core of this phase is to integrate the intervention into everyday practice, relying on the preparatory work started in the initial phase. We decided to follow the approach of the NCCDPHP Knowledge to Action Framework for Public Health (46) and the Stetler Model of Research Utilization (43) in which this phase is called the translation phase. They view the implementation process as translating research into practice. The core of these phases is however similar: it refers to the entire process of putting research into practice (46), thus implementing change into real world settings. In short, the actions that are key within the translation phase are:

1. Introducing the intervention by applying the strategies as defined in the development phase;
2. Monitoring how different components interact with each other to ensure continuous improvement.

All models with a translation phase will agree that key activities within this phase are applying those strategies (30, 45) or types of support (42, 46) that have been defined in the development phase, in order to introduce the intervention. For example, training or coaching is organized (45, 46), leadership- or communication structures are put in place (42, 49), technical assistance is provided or financial resources are made available (46). The Ottawa Model of Health Care Research (30) sees this as a monitoring phase, which means that strategies for introducing and implementing the intervention are to be observed and adjusted if necessary. Within the Research Utilization Model (47), the term “adaptation” is introduced, which means that “*over time, an innovation, the social system into which it is introduced, or both, may change or be modified to facilitate use of the innovation.”* This suggests that interaction is expected between the intervention, the strategies used and the context or setting in which the intervention takes place.

#### Sustainment Phase

Seven process models that we included in our analysis mention some form of sustainment phase. Aarons et al. (22) directly incorporate a sustainment phase and define it as “*the continued use of an innovation in practice*.” This corresponds with what is named the “institutionalization phase” in the NCCDPHP Knowledge to Action Framework for Public Health (46) and the Research Utilization Model (47). Institutionalization of an intervention means that the intended change within an intervention becomes an established activity or norm within the setting it is implemented (46). It becomes integrated into the routines and practices of this setting (47), and it should be ensured that the intervention is applied to all of whom it is aimed (39). Central in the sustainment phase is:

1. Applying the strategies as defined in the development phase to help sustain the intervention;
2. Reflecting upon the actions taken and ensuring continuous improvement.

Indeed, the aim of the sustainment phase of an intervention is that the intended change is maintained and becomes part of the daily routines and practices. This goes beyond a mere adoption of an intervention. The Organizational model for transformational change in health care systems (49) incorporates a similar idea, which is referred to as integration. Blanchard et al. (45) also ^*^speak of integration of new learnings into practice, which they call full implementation. All of these imply that an intended change is adopted and in time harmonizes with, or replaces previously existing practices and activities.

A sustainment phase is also the phase in which continuous improvements ensure a fit between the intervention and the setting in which it is implemented. The Quality Implementation Framework (32) sees the improvement of future applications as the core of this final phase. This is learning from experience. Through reflection and feedback from the setting in which the intervention is introduced, strengths, and weaknesses of the intervention can be detected and acted upon (32). For Blanchard et al. (45) this implies achieving fidelity and improving outcomes. This phase can directly be linked to evaluation, which four process models include as a separate phase.

This notion of continuous improvement can be linked to reflection and evaluation as a part of the process. Several process models include evaluation or measuring performance and outcomes as a phase of the implementation process, for example in the Medical Research Council guidance (38), the CFIR (26), the Ottawa Model of Health Care Research (30), the IOWA model (42), the ACE Star Model of Knowledge Transformation (44), the Stetler Model of Research Utilization (43) and Advancing Understanding of Mechanism of Change in Implementation Science (31), and the model for large scale knowledge translation (39). These frameworks or models generally include minor guidance about how to assess success or failure. There are however also frameworks that are designed specifically to guide the evaluation process, examples of which are the RE-AIM framework (54), the PRECEDE-PROCEED model (55) and the Implementation Outcomes Framework (IOF) (56).

### Framework Components

Throughout the three phases of the implementation process, we distinguish components that have to be taken into account within each phase. Therefore, we looked into what Nilsen (25) calls determinant frameworks. These are designed with the intent to understand and explain what influences implementation outcomes, and thus provide information on which components to focus for implementation success. Some frameworks tend to mainly focus on enlisting relevant context variables [e.g., Theoretical Domains Framework 2.0 (51)], while others also specify the relationships and interactions between types of determinants (25). These frameworks provide valuable input when describing different types of context variables that might hinder or facilitate intervention efforts.

Table 7 gives an overview of how various determinant frameworks refer to the three components that we have extracted: intended change, context and/or strategies. They will provide further guidance on how to understand and work with these elements and how they can affect implementation outcomes.

**Table 7:** Overview of determinant frameworks that incorporate intended change, context, and strategies as components.

**Determinant framework**	**Intended change**	**Context**	**Strategies**
Consolidated Framework for Implementation Research (CFIR), Damschroder et al. (26)	Intervention characteristics	Inner setting Outer setting	-
Integrated Promoting Action Research in Health Services Framework (i-PARiHS), Stetler et al. (33)	Evidence/Evidence and EBP characteristics	Context/Contextual readiness for targeted EBP implementation	Facilitation
CAIMeR theory, Blom amd Morén (52)	Interventions	Contexts	–
Barrier assessment, Cochrane et al. (20)	*Barriers embedded in the guidelines or evidence*	Cognitive-behavioral barriers Attitudinal or rational-emotional barriers Professional barriers Patient barriers System and process barriers	Support or resources
Ecological framework—interactive systems framework for dissemination and implementation, Durlak and DuPre (48)	Characteristics of the innovation	Community level factors Provider characteristics Factors relevant to the prevention delivery system: organizational capacity	Factors related to the prevention support system
Conceptual model for considering the determinants of diffusion, dissemination, and implementation of health service delivery and organization, Greenhalgh et al. (50)	The innovation	System antecedents for innovation System readiness for innovation Outer context	Communication and influence Diffusion and dissemination
Understanding user context framework for knowledge translation, Jacobson et al. (34)	The issue	–	Dissemination strategies
The interdisciplinary conceptual framework of clinicians' compliance with evidence-based guidelines, Gurses et al. (35)	Guideline characteristics	System characteristics Provider characteristics	Implementation characteristics
Four levels of change for improving quality, Ferlie and Shortell (40)	Individual change Group/team change Organizational change Larger system/environment change	–	–
A practical, robust implementation and sustainability model (PRISM), Feldstein and Glasgow (36)	Program (interventions)	External environment Implementation and sustainability infrastructure	-
Translating research into practice, Bradley et al. (41)	Credibility of evidence-based practice	Top-down support Leadership Organizational culture Intervention infrastructure	Coordination of different stakeholders Dissemination Diffusion
Determinants and consequences of implementation effectiveness, Klein and Sorra (37)	–	Climate for Implementation Innovation values fit	Skills/Incentives and disincentives/Absence of obstacles
Conceptual framework, Lau et al. (3)	Intervention	External context Organization Professional	-
Generic Implementation Framework (GIF), Moullin et al. (19)	Innovation	Context domains Factors	Strategies
The ottawa model of health care research, Logan et al. (30)	Evidence-based innovation	Practice environment	Transfer strategies
Theoretical domains framework (v2.0), Atkins et al. (51)	–	*Provides a list of domains that can be incorporated as context variables*.	–
Conceptual model of evidence-based practice implementation in public service sectors, Aarons et al. (22)	–	Outer context Inner context	–

#### Intended Change

The intended change deals with any conscious change into current practices of primary care providers or any actions that actors undertake (57), which are expected to solve a care or quality gap (58). This can take the form of a task-oriented change in practice (33), require behavioral change (59) either at individual or group/team level (40) and/or have a broader organizational impact whereby a more complex transformational change is initiated (33). The intended change derives from the objectives of the intervention, with the assumption that the initiated change will contribute to realizing these objectives (58).

Twelve of the determinant frameworks mention a component similar to the intended change as part of the implementation process. This is referred to as (characteristics of) an intervention (3, 26, 52), innovation (19, 30, 48, 60), change (40), program (36) or issue (34), or involves an evidence based practice (20, 30, 33, 41) or guidelines (20, 35). Determinant frameworks that do not mention the intervention as a separate component either focus on context variables (40), domains (51) or barriers (20), or incorporate intervention aspects in general implementation characteristics (35, 37).

The CFIR (26), the Interactive Systems Framework for Dissemination and Implementation (48) and i-PARiHS specifically zoom in on the characteristics of such an intended change (in these models referred to as intervention, innovation, or evidence-based practice). This indicates that an intervention or intended change is complex, multi-faceted, and different components will be interacting with each other (26). Characteristics that are mentioned are among others compatibility (33, 35, 48), adaptability (36, 48), complexity (26, 33, 35, 36), and/or relative advantage (33, 35). Such inherent characteristics of the intervention will have an impact on its overall implementation success.

As the intended change is expected to contribute to realizing the objectives of the intervention, it is important to define what outcomes are expected from the intended change. Four determinant frameworks incorporate *results* (52), *output* (52), *outcomes* (30), (implementation or innovation) *effectiveness* (37), or *successful implementation* (33) as separate components. This helps focusing on the objectives that are set when defining an intervention and the benefits that arise when implementation is successful (37). The time frame in which results can be observed, can differ majorly. Certain results are obtained early on, while others only exist in the long-term even after the intervention is finished (52). When defining the intended change, it is thus key to not only define the behavioral or organizational change that is expected, but also the expected results and how this can be evaluated.

#### Context

Context variables can be defined as “*the set of circumstances or unique factors that surround a particular implementation effort* (26).” They are dynamic factors that interact, influence, modify, and facilitate or constraint intervention and implementation efforts (53). Context variables are most prominent in what Nilsen (25) defines as determinant frameworks, in which the main objective is to gain insight in those barriers and facilitators that impact implementation outcomes (25). Some are built with the interaction of context variables (40), context domains (51), or barriers (20) as a main focus. Most frameworks indeed incorporate some form of context variables as an essential part of the implementation process. i-PARiHS (33), the Conceptual Model of Evidence-Based Practice Implementation in Public Service Sectors (22), the CFIR (26), the CAIMeR theory (52), and the GIF (19) directly incorporate context, contextual readiness, inner and outer context, context domains, setting, or factors as a component of the framework. A distinction is sometimes made between inner- and outer context or setting (22, 26), which mentions inner context variables as being specific to a person, team our organization (on micro and meso level), while outer context variables are broader in nature such as socio-economic or policy variables (on macro level).

When referring to context, some frameworks only incorporate context variables on the macro level. They zoom in on the so called outer context (50), external context (3), or external environment (36). Elements on an organizational or individual-adopter level are then incorporated under a different name. For example, organizational aspects can also be referred to as system characteristics (35), system antecedents or system readiness for innovation (50), practice environment (30), system and process barriers (20), implementation and sustainability infrastructure (36), organizational culture (41) or climate for implementation (37), intervention infrastructure (41), or factors relevant to the prevention delivery system (48).

When it comes to the micro context, individual adopter characteristics are mentioned by fewer frameworks. They are referred to as professional (3), or provider characteristics (35, 48), or more specifically as cognitive-behavioral barriers, attitudinal, or rational-emotional barriers or professional barriers (20), which indicates that individual adopter characteristics can cover a wide range of micro level aspects. This is also noticeable in the Theoretical Domains Framework (51), in which a wide variety of “domains” is mentioned, many of which are individual adopter characteristics such as professional role, beliefs about capabilities, etc.

On the micro level, context variables highly relate to the actors to which the intended change concerns. Greenhalgh et al. (50) state that “*people are not passive recipients of innovations*.” The dynamic interplay of how individuals relate to the organization in which they work (26) and their general assumptions about people, society and their profession (52) influences their perception and the way in which they make sense of an intended change. Six determinant frameworks include *actor*s (52), *individuals involved* (26), *potential adopters* (30, 50), *recipients* (36), or *the user group* (34) as a core component. Incorporate actors as one of the components strengthens the view that actors have an impact on the way an intervention is realized. In five determinant frameworks, the influence actors have on implementation success is recognized by including individual attitudes, cognitions, or professional characteristics as a context variable (3, 20, 35, 48, 51). The component actors can thus be incorporated as a separate component of an implementation model, but it can also be included as a micro level context variable.

Overall, there is a wide belief that the context in which a primary care intervention takes place highly determines implementation success (10). This makes scanning and taking into account the context key for each phase of the implementation process. When determining implementation strategies, context variables must be taken into account in order for strategies to be tailored and fit local circumstances (10). This is in line with realist evaluation, whereby the general aim is to find out “*what works, for whom, and under what conditions?”* (6). In this approach, context variables are the conditions in which an intervention takes place.

#### Strategies

Implementation strategies can be defined as the approach(es) and means that are used to ensure or enhance the adoption of the target behaviors and other requirements of the primary care intervention by the targeted actors (10, 61). Whereas, the intended change refers to *what* is to be implemented, the strategies refer to *how* they are to be implemented and is linked to the process or mechanism that intervention designers want to trigger in order to accomplish implementation.

Implementation strategies are directly referred to in few process models, such as Advancing Understanding of Mechanism of Change in Implementation Science (31), whereby a first step to implementation is to specify the implementation strategies; the Ottawa Model of Health Care Research (30) in which transferring strategies is a part of monitoring the uptake of the intervention and in the GIF (19), in which the strategies are viewed as the approaches to respond to barriers and facilitators. Throughout other determinant frameworks, a component similar to implementation strategies is included in eight of the models we included in our analysis, either in the form of facilitation (33), support (e.g., training, assistance) (20, 48), implementation characteristics (35) and dissemination and/or diffusion of strategies (34, 41, 50). Frameworks also tend to incorporate those elements that are considered to be most decisive as strategies, such as communicational aspects (50), coordination of different stakeholders (41), or the use of incentives and disincentives (37).

Implementation strategies are discussed more in-depth in the Expert Recommendations for Implementing Change (ERIC) study, in which a compilation of 73 implementation strategies was made (62, 63). This can serve as a guide for when the most fitting implementation strategies have to be selected for the implementation of a certain intervention. To make more sense of the wide diversity of implementation strategies, they often are categorized. For example, Powell et al. (64) distinguishes between strategies that are related to either planning, educating, financing, restructuring, managing quality, and/or attending to policy context. Another categorization can be found in Charif et al. (65), who differentiate strategies that are related to either the health infrastructure, policy and regulation, financing, human resource, or patients (65).

Implementation strategies can be very different depending on the type of change that is initiated, and should ideally be tailored to fit the inner and outer context (10, 66), making use of the facilitators or barriers that are observed in order to ensure a fit between the intervention and its context (3). When defining implementation strategies to implement one's intervention, Proctor et al. (67) have set up guiding principles to name, define, and operationalize implementation strategies by firstly specifying the following elements: (1) actor, (2) action, (3) action target, (4) temporality, (5) dose, (6) implementation outcome affected, and (7) justification. These can support intervention designers in defining implementation strategies.

In short, implementation strategies are expected to lead to an intended change in a given context. This means that there is an underlying process that will bring about this change. Three determinant frameworks include this *(implementation) process* (26, 60) or *mechanism* (52) as one of the core components. These frameworks have a more explanatory approach and put more emphasis on understanding the process of change. For complex interventions, this consists of many interdependent sub-processes that may or may not follow a clear path to success (26). The process involves decision making activities, the use of resources, communication, and collaboration (50). Blom and Morén (52) view this as an either social, socio-psychological, or psychological mechanism that is at the base of change. Greenhalgh et al. (50) and Lewis et al. (31) also refer to *linkages* or *effect modifiers* and Aarons et al. (22) speak about *interconnections*, referring to the fit between an innovation and a system or organization that comes into play when introducing a change. These frameworks thus incorporate the process or mechanism of change as a core element that needs to be understood in order to fully know how to target certain interventions in specific settings. When choosing implementation strategies, it is thus recommended to make explicit the assumptions of how a certain strategy will lead to the intended change in a given context.

## Discussion

We have identified the core building blocks of an overarching implementation framework for complex interventions in primary care services. Throughout our narrative review, three core phases are detracted that describe the process of implementation in relation to three core components. This process can roughly be divided in a development phase, a translation phase, and a sustainment phase. For each phase, three main components are essential to define, tailor, and manage to successfully implement an intervention in a specific setting. These are the intended change, the context, and the implementation strategies. Other related components that are closely linked to these three components may still be relevant, such as actors, the process or mechanism, and the outcomes and evaluation of the intervention.

An overarching implementation framework is needed to transcend the solely theoretical models and to aim for a model that is both explanatory as well as actionable. Context variables should be given a prominent place in this, as tailoring interventions to local circumstances is considered key for reaching implementation success (9, 10). By focusing on the core components intended change, context, and strategies we propose meaningful concepts to intervention designers and practitioners for reflecting upon the interactions of these components. The next step is synthesizing these core building blocks into a framework that consists of a clear and actionable pathway for intervention designers, and which enables them to prioritize and reflect upon those actions that need to be taken for the implementation of complex interventions.

Our research is part of a larger project that intends to make progress in three main research areas: to improve goal oriented care, self-management, and inter-professional collaboration. In each of the three areas, one or more interventions will be used for developing and evaluating the implementation of interventions in these three areas. The model that we will further develop will allow to develop and implement interventions with broad consideration of the setting or context in which they will be introduced, and how this interacts with the intended change and the implementation strategies that are used.

A limitation of our review is that we did not gather and include our sources in a systematic way. We used a more intuitive approach whereby sources were gathered mainly through expertise from our research team, by database searches with a set of different key words and by further use of a snowball approach that lead to the most prominent frameworks and models that exist. Furthermore, as we have only included English literature, there seems to be a slight overrepresentation of literature deriving from native English authors and/or institutions. Moreover, we have no view on gray literature or literature written in foreign languages, which might further limit our scope.

Although there is no assurance that we have covered all relevant literature, the methodology of a narrative review allowed us to explore the broad range of implementation literature and interpret various approaches in the light of interventions that aim toward pro-active, person-centered primary care. This way, we could harmonize literature into insightful constructs and phases which are to be made concrete when further applying them in the defining and execution of interventions.

## Conclusion

An overarching implementation model is needed to bridge the gap between scientific evidence and actual practice in primary care. Through a narrative review, we have identified the core building blocks that form the common thread of existing implementation frameworks or models and we synthesized it in three core phases (a development phase, a translation phase and a sustainment phase) and three core components (the intended change, the context and the implementation strategies). These core building blocks can be used to develop an overarching implementation model that is both explanatory, as well as actionable. The main phases and components are the basis on which further guidance for intervention designers will be elaborated. A strength of the model that we will develop based upon this research is that it will be further developed and refined in collaboration with three research teams that will actively use the model to develop and introduce one or more interventions in primary care. This allows for direct feedback on its applicability and therefore ensures its actionability.

## Author Contributions

IH wrote the main manuscript text. AD, EV, PR, and SA contributed to the different steps of the making of this manuscript. All authors reviewed the manuscript.

## Conflict of Interest

The authors declare that the research was conducted in the absence of any commercial or financial relationships that could be construed as a potential conflict of interest.

## Publisher's Note

All claims expressed in this article are solely those of the authors and do not necessarily represent those of their affiliated organizations, or those of the publisher, the editors and the reviewers. Any product that may be evaluated in this article, or claim that may be made by its manufacturer, is not guaranteed or endorsed by the publisher.

## Footnotes

^1^World Health Organization. (2008). Primary care, now more than ever. https://www.who.int/whr/2008/whr08_en.pdf (accessed May 15, 2020).
